# Correction: Patsaki et al. Benefits from Incorporating Virtual Reality in Pulmonary Rehabilitation of COPD Patients: A Systematic Review and Meta-Analysis. *Adv. Respir. Med.* 2023, *91*, 324–336

**DOI:** 10.3390/arm92010011

**Published:** 2024-02-01

**Authors:** Irini Patsaki, Vasiliki Avgeri, Theodora Rigoulia, Theodoros Zekis, George A. Koumantakis, Eirini Grammatopoulou

**Affiliations:** Department of Physiotherapy, University of West Attica, 11521 Athens, Greece

**Error in Figure 3**

In the original publication [1], there was a mistake in “Figure 3 Forest plot showing the effects of VR-Training on the 6MWT [24,25,27,28]”, due to the fact that the data were placed in reverse for the study of Suntanto et al., 2019; i.e., by mistake, the control group data were entered in place of the experimental group data. The meta-analysis results were different; therefore, the scientific conclusions were affected to a minor degree, and appropriate changes were made in the text as well.

**Figure 3:**
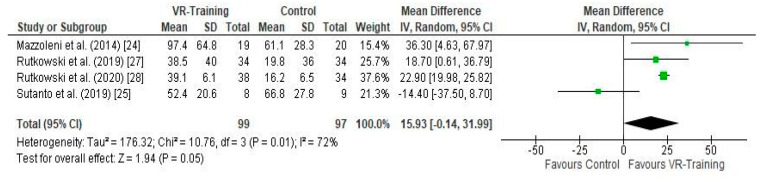
Forest plot showing the effects of VR-Training on the 6MWT [24,25,27,28].

**Error in Figure 5**

In the original publication, there was a mistake in “Figure 5 Forest plot showing the effects of VR-Training on the MRC dyspnea scale [24,25]”, due to the fact that the data were placed in reverse for the study of Suntanto et al., 2019; i.e., by mistake, the control group data were entered in place of the experimental group data. The meta-analysis results were only slightly different; therefore, the scientific conclusions were unaffected.

**Figure 5:**
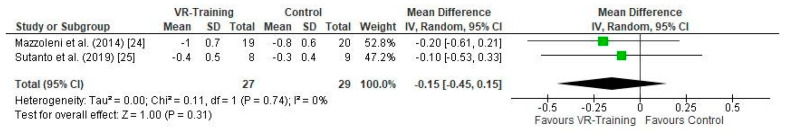
Forest plot showing the effects of VR-Training on the MRC dyspnea scale [24,25].

**Text Corrections**

There was an error in the original publication. “The variables examined were the aerobic capacity for exercise, lung function, and anxiety and depression, with significant improvement regarding 6MWT and FEV1 (*p* < 0.05)”.

A correction has been made to the “Abstract”: “Chronic Obstructive Pulmonary Disease (COPD) is characterized by irreversible airflow limitation. Patient participation in Pulmonary Rehabilitation (PR) programs has a beneficial effect on disease management, improving patients’ functional capacity and quality of life. As an alternative to traditional programs or as a complementary activity, the inclusion of virtual reality (VR) games is proposed. The aim of this research study was to investigate the effectiveness of incorporating VR in the pulmonary rehabilitation program of patients with COPD. A systematic literature search was performed for randomized controlled trials (RCTs) in the electronic databases Google Scholar, PubMed and Pedro from January 2014 to March 2022. The search involved screening for studies examining the effectiveness of enhancing PR with VR. The PEDro (Physiotherapy Evidence Database) scale was chosen as the tool to assess the quality of studies. A meta-analysis was performed where possible. Six studies were included in this systematic review. The PEDro scale showed five studies of good methodological quality and one of fair quality. The variables examined were: aerobic capacity for exercise, lung function, anxiety and depression, with non-significant improvement for the MRC Dyspnea scale, marginally non-significant improvement regarding 6MWT (*p* = 0.05) and significant improvement for FEV1 (*p* < 0.05). There was variability noted in the VR applications and the proposed rehabilitation that the experimental groups followed. The application of VR is recommended in COPD patients, in combination with conventional PR. VR was found effective in increasing the therapeutic effect and should be considered as a mean of increasing accessibility to PR. Therefore, further research, as well as additional RCTs regarding the effectiveness of VR in patients with COPD, seem necessary”.

There was an error in the original publication. “A mean difference (MD) (95% CI) = 22.7 (19.92 to 25.63) m, favoring VR training with statistical significance (Z = 15.66, *p* < 0.001) and no statistical heterogeneity (I^2^ = 0, *p* = 0.7), was noted, based on a 7/10 Pedro quality score on average (Table 1)”.

A correction has been made to “3.5. Effects of Interventions”, “3.5.1. Effect of VR-training on Exercise capacity (Figure 3)”:

“The effect of VR-Training with or without other parallel interventions on 6MWT, calculated in meters, was evaluated in four studies [24,25,27,28], including 196 participants in total (Figure 3). A mean difference (MD) (95% CI) = 15.93 (−0.14 to 31.99) m, favoring VR-Training with marginal non-statistical significance (Z = 1.94, *p* = 0.05) and substantial heterogeneity (I^2^ = 72%, *p* = 0.01) was noted, based on an 8/10 Pedro quality score on average (Table 1)”.

There was an error in the original publication: “A mean difference (MD) (95% CI) of −0.06 (−0.36 to 0.24) was found, with one study favoring VR-Training and the other having the opposite effect, did not show statistical significance overall (Z = 0.38, *p* = 0.7). However, no statistical heterogeneity (I^2^ = 0, *p* = 0.32) was noted based on Pedro quality evidence”.

A correction has been made to “3.5. Effects of Interventions”, “3.5.3. Effect of VR-Training on Subjective Dyspnea (Figure 5)”: “The effect of VR-Training with or without other parallel interventions on the MRC dyspnea scale was evaluated by two studies [24,25] including 56 participants in total (Figure 5). A mean difference (MD) (95% CI) of −0.15 (−0.45 to 0.15) was found, with both studies favoring VR-Training, but overall not reporting statistical significance (Z = 1.00, *p* = 0.31); however, no statistical heterogeneity (I^2^ = 0%, *p* = 0.74) was noted based on 5/10 Pedro quality evidence on average (Table 1)”.

There was an error in the original publication. “One of the main findings from this review was the positive effect that these novel technologies have on exercise capacity”. and “It is well-presented that AVG could induce high physiological demands capable of producing significant training effects if used regularly as a training method [31] (Kuys et al., 2011)”.

A correction has been made to “4. Discussion”, second paragraph: “One of the main findings from this review was the marginal positive effect that these novel technologies have on exercise capacity. The heterogeneity noted among the training modalities could have limited the overall effect. Yet, this is a key outcome that is measured in all respiratory patients after completing a PR program. There were three studies that showed significant difference among experimental groups in 6MWT [24,27,28], with all managing to exceed the minimal clinical importance difference of 35 m. All three included games that were focused on improving dynamic balance, strengthening the lower and upper limbs and improving endurance, thus providing an extra stimulus. Only [28] Rutkowski et al. did explore and showed that even the use of Kinect-based training alone could lead to a significant improvement of 6MWT. It could be assumed that AVG could induce high physiological demands capable of producing significant training effects if used regularly as a training method [31] (Kuys et al., 2011). In the studies included in this systematic review, the intensity of training was monitored using the Borg dyspnea scale and ranged in similar values (4–6 points) as recommended by the ATS (American Thoracic Society) guidelines for pulmonary rehabilitation for COPD patients [32]. LeGear et al. [33] managed to show that the level of physical effort produced during VR rehabilitation with Nintedo Wii was similar to that produced during training on a treadmill. With the exception of one study [15], all the others tried to incorporate either a strengthening or an endurance training component or even both through the gaming intervention, following the ATS guidelines regarding COPD rehabilitation. Even the ability to provide a training session safely is of significant importance. We should bear in mind that a well-designed program tailored to the abilities and needs of the patients even when games are utilized as part of the training regimen could have positive results. Being able to improve exercise capacity and thus increasing physical activity is a key component to reducing exercise intolerance and providing a healthier and more active lifestyle for this population. Also, the advantages that gaming brings in motivation, engagement and pleasure are important components of any exercise-based intervention. Additionally, we should add the ability of delivering this service remotely, thus overcoming a lot of barriers that patients mention when dropping out of PR [34]. Similar results were presented by a recent meta-analysis in which non-English studies were included [35]”.

There was an error in the original publication. “This systematic review and meta-analysis demonstrated that VR programs could be used to augment the therapeutic effect of PR in COPD patients, increasing exercise capacity and having a beneficial effect on lung function”.

A correction has been made to “6. Conclusions”:

“This systematic review and meta-analysis demonstrated that a VR program could be used to augment the therapeutic effect of PR in COPD patients, as it seems to have a beneficial effect in exercise capacity and on lung function. It is a safe and well tolerated intervention that offers adequate work loading to traditional PR, while being delivered at home and in one’s spare time. Gamification features add enjoyment and create a spirit of competition that fuels the ongoing engagement. Further studies are needed to evaluate the effect of VR programs on other significant variables related to COPD wellbeing such as depression, anxiety, cognition and quality of life. Assessing the long-term utilization of these programs and maintenance of positive results will be of high importance for this population and those with other respiratory conditions”.

The authors state that the scientific conclusions are unaffected. This correction was approved by the Academic Editor. The original publication has also been updated.

## References

[1] Patsaki I., Avgeri V., Rigoulia T., Zekis T., Koumantakis G.A., Grammatopoulou E. (2023). Benefits from Incorporating Virtual Reality in Pulmonary Rehabilitation of COPD Patients: A Systematic Review and Meta-Analysis. Adv. Respir. Med..

